# Grassland ecosystem responses to climate change and human activities within the Three-River Headwaters region of China

**DOI:** 10.1038/s41598-018-27150-5

**Published:** 2018-06-13

**Authors:** Ze Han, Wei Song, Xiangzheng Deng, Xinliang Xu

**Affiliations:** 1 Key Laboratory of Land Surface Pattern and Simulation, Institute of Geographic Sciences and Natural Resources Research, Chinese Academy of Sciences, Beijing, 100101 China; 2 Center for Chinese Agricultural Policy, Chinese Academy of Sciences, Beijing, 100101 China; 3 State Key Lab of Resources and Environmental Information System, Institute of Geographical Sciences and Natural Resources Research, Chinese Academy of Sciences, Beijing, 100101 China

## Abstract

The Three-River Headwaters region (TRHR) of China is an important part of the Qinghai-Tibetan Plateau. Although the TRHR is rich in grassland resources, the ecosystem of this area is extremely fragile. Natural and artificial interference have been key to the development of grassland ecosystem spatiotemporal heterogeneity, although the intensity and mode of their influence on ecological processes varies depending on scale; analyses in this area are therefore also scale-dependent. We use multi-scale nested data to analyze the mechanisms underlying the influence of climate change and human activities on grassland net primary productivity (NPP) by applying a multi-level modeling approach. The results of this study show that: (1) The annual grassland NPP of the TRHR has risen in a wavelike pattern over time, increasing by 39.88% overall; (2) Differences of 54.9% and 41.1% in temporal grassland NPP can be attributed to variations between these watersheds as well as county characteristics, and; (3) Although the ‘warm and moist’ climate trend seen over the course of this study has proved beneficial in enhancing grassland NPP, the rate of increase has tended to be faster in relatively dry and warm regions. Economic development and population growth have both exerted negative impacts on grassland NPP.

## Introduction

Grassland ecosystems are important components of ecological communities on Earth^1,2^, and perform key functions in carbon (C) cycling, climate regulation, and the maintenance of biological diversity^3–6^. However, since the middle of the 20^th^ century, these ecosystems have been subjected to major environmental fluctuations because of climate change and intensified human activities^7–10^. These ongoing changes have affected grass growth and the functioning of constituent ecosystem^11,12^. In this context, net primary productivity (NPP) is one key indicator that can be used to measure the ability of a grassland ecosystem to maintain a level of sustainable development^13^, recorded via the net amount of C captured by land plants annually through photosynthesis^14^. Variations in NPP are therefore indicative of relationships between vegetation growth and the surrounding environment^15,16^; it is therefore critical to better understand the responses of grassland NPP to both climate change and human activities.

The approaches that are currently used to reveal the relationship between grassland NPP and underlying driving factors include field-based experiments^17,18^, trend tests^12,19^, and ecosystem modeling^20^, and usually integrate ecological processes against the same background. However, because both natural and anthropogenic disturbances are spatially heterogeneous, a number of previous studies have demonstrated that ecological phenomena tend to manifest in the form of multi-level and multi-hierarchy structures^21,22^; this means that both the intensity and mode of a disturbance that results from either natural or anthropogenic factors will exhibit different characteristics depending on scale^23–26^ and that there will be interactions between these different hierarchical levels^27^. At the microscopic scale, for example, grassland plant populations with similar biological characteristics tend to survive in similar habitats, while differences in environmental conditions can lead to spatiotemporal species variations. In contrast, the development of grasslands at the macroscopic scale depends not only on climate and geographical characteristics^28^, but also on interventions by conservation and management policies (e.g. prohibition of enclosed grazing and the construction of ecological reserves). These interventions can themselves markedly alter the population characteristics of grasslands, including their homogeneity, richness, and diversity^29–32^. Ignoring spatiotemporal heterogeneity will therefore lead to inaccurate results as group effects are disregarded.

**Figure 1:**
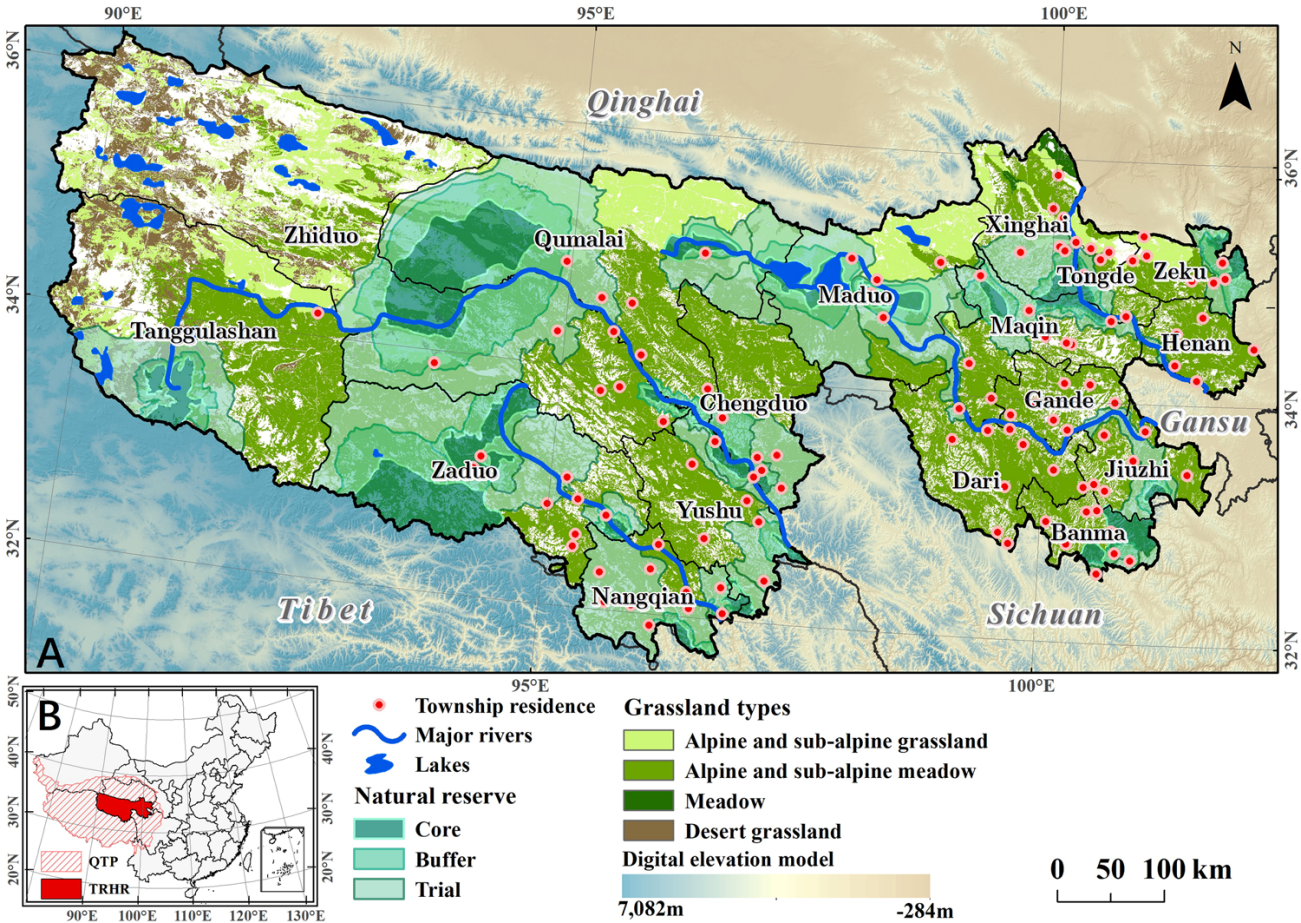
Maps to show the location of study sites. (**A**) Distribution of grassland types and national nature reserves (NNRs) within the TRHR. (**B**) The QTP and the TRHR. The natural reserve of the TRHR contains three zones that are referred to as core, buffer, and trial areas. As the first of these is strictly managed with no grazing allowed and with measures implemented to protect endangered species, all development and land use is prohibited; all former residents of this core zone have been resettled elsewhere as this is a ‘no man zone’ area. Conservation is also promoted within the buffer zone in tandem with limited and rotational grazing, while the trial zone area is used for scientific investigations, ecotourism, and other green industries^40,88^. The digital elevation model (DEM) data with ~30 m resolution used here (**A**) was obtained from NASA Shuttle Radar Topography Mission Version 3.0 Global 1 arc second dataset (https://earthexplorer.usgs.gov/)^89^. The boundary data of QTP, TRHR, and China administrative regions used here (**B**) were provided by Global Change Research Data Publishing & Repository (http://www.geodoi.ac.cn/weben/doi.aspx?id=135)^90^, Xu^35^, and the Data Center for Resources and Environmental Sciences, Chinese Academy of Sciences (http://www.resdc.cn/data.aspx?DATAID=200)^91^, respectively. These maps were created using the software ArcMap 10.2.2 (http://www.esri.com/software/arcgis/arcgis-for-desktop)^92^.

The Three-River Headwaters region (TRHR) of China is located in the northeastern area of the Qinghai-Tibetan Plateau (QTP) and is the birthplace of the Yangtze, Yellow, and Lancang rivers. This region is also known as ‘Asia’s water tank’^33^ as it comprises the hinterland and main body of the QTP (Fig. 1). Grasslands are the major components of ecological environments in the TRHR and provide numerous goods and services that are valuable to humans including soil conservation, C sequestration, and water retention. At the same time, the harsh environments that characterize this region mean that single and continuous alpine meadows are the dominant surface covering layer^34^; this has resulted in a fragile ecological environment which is highly sensitive to changing climatic conditions^33,35,36^. A large body of recent work has demonstrated that the climate of this region has conformed to a ‘warm and moist’ trend over at least the last 50 years^33,37^, a situation which has proved highly beneficial for increasing grassland NPP^38^. However, as the agricultural economy of the TRHR has rapidly developed since the 1980s, human impacts such as overgrazing, grassland abandonment, and construction have all interfered with ecological processes and caused grassland degradation, habitat loss, and landscape fragmentation^38,39^. These impacts have also markedly affected the ecological welfare of residents downstream^40^.

A series of grassland restoration projects have been implemented within the TRHR since 2000 to protect this resource, including grazing bans and rotational systems, retiring livestock, and restoring grassland. The Chinese government also established the second-largest nature reserve in the world within the TRHR, the Sanjiangyuan National Nature Reserve (SNNR)^41,42^, as well as the largest ecological project nationally, the Ecological Protection and Restoration Program (EPRP)^43,44^ in 2003 and 2005, respectively. Some previous research has shown that the implementation of these projects has contributed to grassland ecosystem restoration, in particular controlled degradation^45^ as well as increased yields^43^ and NPP^42,46^.

As animal husbandry in the TRHR is important for livelihoods, the growth of grasslands not only impacts the ecological situation and the development of these practices regionally, but also influences ecological security and the economic development of downstream regions as well as across the whole of China. It is therefore critical to determine how grassland production in the TRHR is likely to change given the joint influence of climate and human activities (i.e. population levels, the economy, and animal husbandry) as well as the probable extent of this variation. Current related research in this area has mainly focused on grassland productivity response mechanisms to climate change, while human activities have seldom been addressed. Indeed, in terms of research scale, previous work has also tended to ignore the nested relationships between ecological phenomena and interactions between different scales. The objectives of this study are therefore to: (1) Investigate changes in grassland land use patterns within the TRHR between 1988 and 2012; (2) Reveal trends in NPP changes within this region, and; (3) Establish a multi-level linear model (MLM) to explore the responses of grassland NPP to both climate change and human activities at the level of counties and small watersheds.

## Results

### Spatiotemporal variation in the NPP of different grassland types between 1988 and 2012

**Figure 2:**
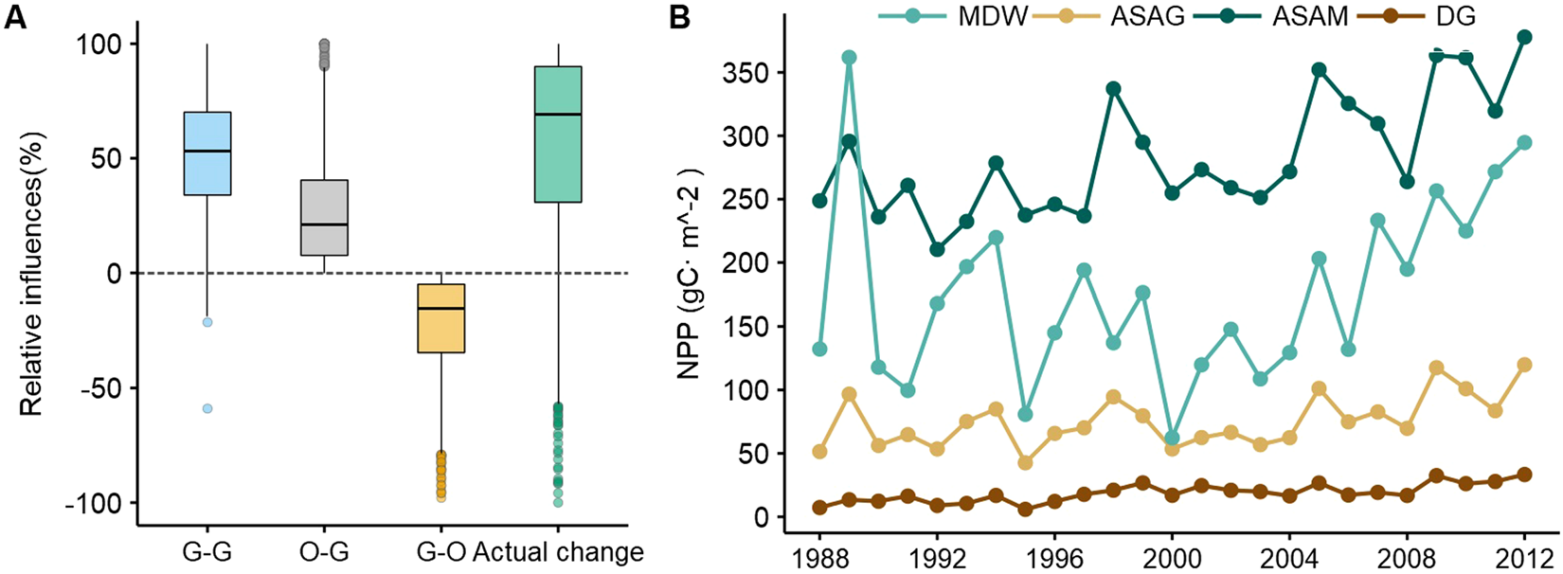
Trends in mean annual grassland NPP values between 1988 and 2012 within the TRHR. (**A**) Average values and standard deviations (SD) of different grassland use type impacts on NPP changes. Conversions denoted G-G, O-G, and G-O refer to the non-conversion of grassland (G-G), conversion from other land uses (O-G), and conversion to other land uses (G-O), respectively. (**B**) Linear trends in annual NPP of different retained unconverted grassland types. The dashed line on this graph is the linear regression fit to annual NPP. Grassland types denoted MDW, ASAG, ASAM and DG refer to the meadow (MDW), alpine and sub-alpine grassland (ASAG), alpine and sub-alpine meadow (ASAM) and desert grassland (DG), respectively.

The total amount of grassland NPP recorded in the TRHR increased from 49.18 tC to 73.51 tC, between 1988 and 2012, a net increase of 24.53 tC. This corresponds to a mean grassland NPP increase from 256.29 gC m^−2^ to 358.50 gC m^−2^, an increase of 39.88% at an average annual growth rate of 1.60%. Statistical analyses of land use (Figs S1, S2, Table S1) and NPP changes within each small watershed (Fig. S3) show that variation in values of the latter for retained unconverted grassland account for 51% of overall average increases, while changes due to grassland conversion account for just 7%, including the positive effect of conversion to this land use type (26%) and the negative effect of conversion into others (−19%) (Fig. 2A).

Data show that grassland NPP values within the TRHR are related to different grassland types. Thus, per unit area NPP values for alpine and sub-alpine meadows (ASAM) and meadows (MDW) were both higher than those for alpine and sub-alpine grasslands (ASAG) and desert grasslands (DG), at average values of 283.97 gC m^−2^ and 176.36 gC m^−2^, respectively. The latter grassland category (DG) is characterized by the lowest per unit area average NPP values (18.68 gC m^−2^), while the total values for meadows were lowest overall, just 0.19 tC yr^−1^. The average total NPP values for alpine and sub-alpine grasslands as well as desert grasslands were higher, at values of 0.34 tC yr^−1^ and 0.19 tC yr^−1^, respectively.

A clear temporal pattern in retained unconverted grassland NPP values is evident in terms of interannual variations. Data show that between 1988 and 2012, recorded per unit area NPP values for different grassland types all showed significant interannual increases as well as large differences in their degrees of fluctuation, especially over the last decade between 2000 and 2012) (Fig. 2). Categories of ASAM and DG exhibited smaller fluctuations over this period while since the area covered by MDW remained relatively small (just 0.42% of total retained unconverted grassland), variation of this grassland type was both the largest and most unstable.

**Figure 3:**
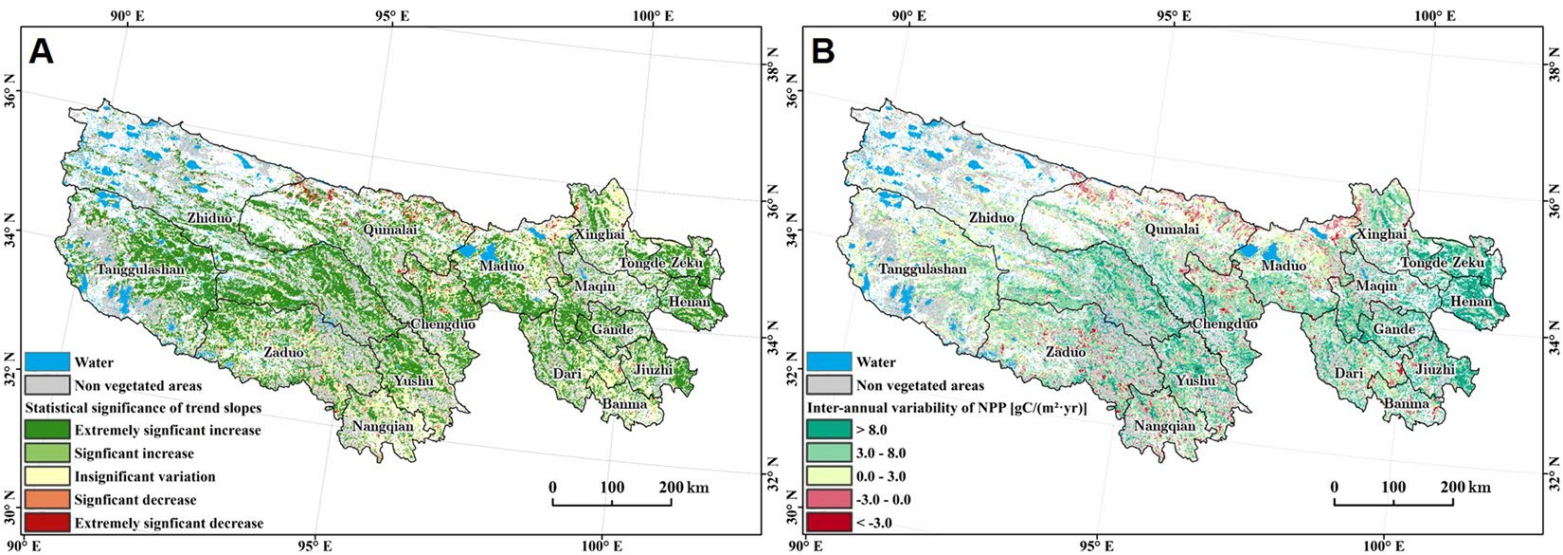
Maps to show spatial trends in annual mean grassland NPP values across the TRHR between 1988 and 2012. (**A**) The statistical significance of trends. Note that NPP data for 2000 and 2012 for the TRHR were obtained from Xu^35^; these data were generated using the global production efficiency model incorporated at a localized level. (**B**) Inter-annual variability in grassland NPP between 1988 and 2012. These maps were created using the software ArcMap 10.2.2 (http://www.esri.com/software/arcgis/arcgis-for-desktop)^92^.

Data show that the distribution of NPP varies depending on grassland type and climatic gradient (i.e. precipitation and temperature). Grassland NPP values across the TRHR varied between 6.07 gC·m^−2^·yr^−1^ and 1,086.87 gC·m^−2^·yr^−1^ over the course of this study; indeed, NPP values in the east and south of this region exhibited relatively large inter-annual variations between 1988 and 2012 and have which tended to increase over the last 25 years. Significant increases in grassland NPP have been recorded in most areas of the TRHR, encompassing 63.22% of the total area covered by this ecosystem type (Fig. 3A). A total area of 8,200 km^2^ across the TRHR has experienced a significant increase in NPP, encompassing 4.48% of total grassland, while values have decreased overall in some regions, mainly within Qumarleb, Zaduo, Nangqian, and Maduo counties (Fig. 3B). In these regions, areas characterized by highly significant reductions in NPP cover 7,690 km^2^, encompassing 3.91% of total grassland area, while zones characterized by significant decreases encompass 1,260 km^2^ (0.68% of total area). Regions that have experienced no obvious changes cover about 27.7% of total grassland area.

### Responses in the distribution of grassland NPP to climate change and human activities

We utilized a three-level growth model to investigate the scale of grassland NPP decomposition across the TRHR, evaluating 1,817 small watersheds within 18 counties (Fig. S2). Variance estimation results based on a random unconditional model (Table 1) show that intraclass correlation coefficients (ICC) at the level of small watersheds (ICC_Watershed_ID_) and counties (ICC_County_ID_) in Model 1 are 0.549 and 0.411, respectively. These results show that statistically significant variation in grassland NPP has occurred over time in the intercepts and rates of change grassland NPP values at both levels.

**Table 1:** Unconditional models used for model selection.

	log(NPP)
	*Estimate* (*CI*)
**Fixed parts**	
Intercept	5.135*** (4.59–5.68)
Year	−0.000 (−0.01–0.01)
Year^2^	0.001*** (0.00–0.00)
**Random parts**	
σ^2^	0.160
τ_00, Watershed_ID_	1.781
τ_00, County_ID_	1.354
N_Watershed_ID_	1,817
N_County_ID_	18
ICC_Watershed_ID_	0.541***
ICC_County_ID_	0.411***
Observations	10,663
R^2^	0.932
Akaike Information Criterion	17,554.005

Notes: (1) t statistics in parentheses; (2) ***^,^ ** and *denote 1%, 5%, and 10% significance levels, respectively.

Compared with their unconditional counterparts, our step-by-step conditional models include a range of different explanatory variables (i.e. climate, grassland, soil, and socioeconomic variables) to capture a proportion of variance (Table S2). Results show that between-group variance at both small watershed and county levels (τ_00_) markedly declined between Model 1 and Model 6 while within-group variation also decreased from 0.160 in Model 1 to 0.088 in Model 6. These results suggest that most grassland NPP variation is captured by these explanatory variables.

We utilized the fixed part of a three-level growth model to further evaluate decomposition distribution patterns and the influences of grassland NPP. We therefore assessed grassland NPP six times between 1988 and 2012 for each of the 1,817 small watersheds considered in this study at an average interval of 4.8 years. The fit of these unconditional growth models illustrates a slight estimated positive coefficient of quadratic NPP rates of change that differ significantly from zero, while average rates of change were negative and not significant; these data indicate that grassland NPP within the TRHR initially decreased and then increased over the time period of this analysis.

**Figure 4:**
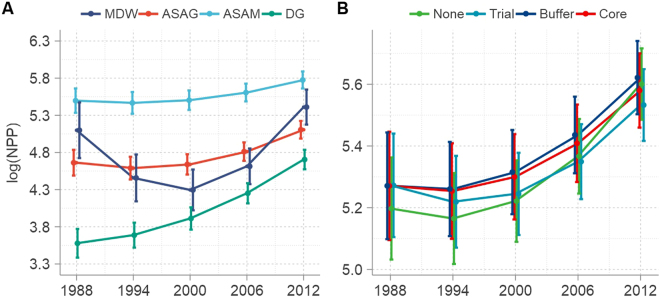
Regression-predicted annual changes in grassland NPP between 1988 and 2012. (**A**) Different grassland types. The definitions of MDW, ASAG, ASAM and DG used here are the same as in Fig. 2. (**B**) NNRs. Curves in this case were fitted using a conditional three-level growth model (Table S2, Model 6). The definitions of core, buffer, and trial zones used here are the same as in Fig. 1A.

We included grassland types and NNRs as dummy variables in our models to distinguish the NPP change trajectories of different ecosystems (Table S2, Models 3 to 6). This analysis revealed that differences among nonlinear NPP change trajectories for different grassland types are evident over time (Fig. 4A); values for DG have steadily increased over the last 25 years at an average annual rate of 4.52%, while values for ASAG, ASAM, and MDW all initially decreased significantly before subsequently rising again. The initial average rates of decrease in these three cases were 1.2%, 0.9%, and 14.5%, before steeper increases were then seen after 2000. In contrast, our results for NNRs reveal that these areas exerted non-significant impact on grassland NPP trajectories over time (p > 0.14) (Fig. 4B); average grassland NPP values on NNRs were 11.18 gC/m^2^ higher than in other regions at the start of this analysis before decreasing within these protected regions at higher rates than outside up until 2000. Data clearly reveal a turning point in grassland NPP trajectories within the TRHR from 2000 onwards.

**Figure 5:**
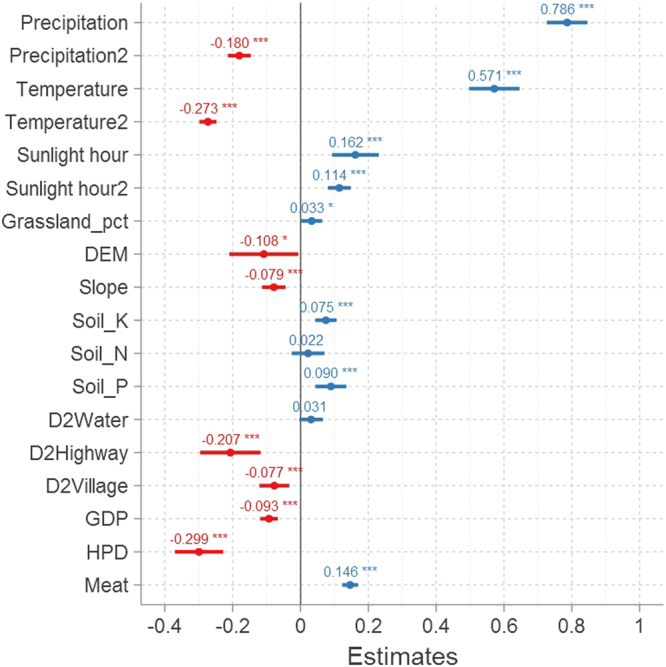
The main effects used within a three-level growth model to predict grassland NPP. All coefficients were standardized in terms of proportional change in grassland NPP with respect to one SD change in precipitation, temperature, sunlight, grassland area percentage (grassland_pct), DEM, slope, soil nutrient percentage (i.e. potassium, K, nitrogen, N, phosphorus, P), the average distance from the small watershed to the nearest water source (D2Water), highway (D2Highway), and village (D2Village), as well as gross domestic product (GDP), human population density (HPD), and meat production (Meat). The values used for this analysis were: precipitation, 158.5 mm; temperature, 2.28 °C; sunlight, 201.05 h; percentage of grassland, 23.99%; DEM, 395.18 m; slope, 5.8°; soil K, 37.17%; soil N, 0.17%; soil P, 0.01%; D2Water, 28.12 km; D2Highway, 196.65 km; D2Village, 55.76 km; GDP: 149 million CNY; HPD, 30.6 thousand people·km^−2^; Meat, 2.66 thousand tons. The t-statistics are in the parenthesis. ***^,^ ** and * denote 1%, 5% and 10% significant level. The solid line in this figure marks the 95% confidence interval (CI) for the estimated effect of variables at the three-level model; thus, those that do not overlap this line can be considered significantly different from zero. The dots denote point estimates for parameter values^47,48,93^. This figure was created using the software R (http://www.Rproject.org/)^94^ and the library sjPlot^93^.

The fixed effect coefficients used in our three-level growth model reveal proportional variations in grassland NPP in response to one SD change in an explanatory factor (Fig. 5 and Table S2, Model 6). These results therefore show that trajectories of grassland NPP change have been significantly dependent on climate (Table S2, Model 6). Indeed, quadratic coefficients for both precipitation and temperature are negative in this model which indicates that change in grassland NPP has been hump-shaped in response to these factors, while the coefficients for both these variables were positive versus productivity; values for these variables initially rose in a linear fashion and peaked at high values. In contrast, estimates for grassland NPP change in response to the number of sunlight hours (shour) exhibited both linear and quadratic positive coefficients; these results imply that increases in the number of sunlight hours have likely been beneficial to grassland NPP growth within the TRHR.

The results of this analysis show that the characteristics of small watersheds, including grassland area percentage, topography (i.e. DEM and slope), soil nutrient content (i.e. N, P, and K), and location, all determine the intercept components of grassland NPP. Calculations reveal a coefficient of 0.033 (*P* = 0.035) for grassland area percentage; this indicates that when the grassland area percentage of a small watershed increases by about 24%, average NPP will also rise by about 3.3%. In contrast, topographic variables (i.e. DEM and slope) have had a mainly negative influence on grassland NPP, causing a gradual decline with increases in altitude and slope. Soil nutrients, however, are positively associated with grassland NPP; a higher soil nutrient level is clearly beneficial to grassland growth. Similarly, the density distribution of highways and villages is also positively related to higher grassland NPP, while the opposite is the case in terms of distance to water sources.

The results of this analysis show that human activities (i.e. meat production, economic growth, and HPD) at the county scale have also significantly affected grassland NPP changes. Estimations show that both GDP and HPD have had a negative influence on NPP, with coefficients of −0.093 (*P* < 0.001) and −0.299 (*P* < 0.001), respectively, while meat production (meat) has had a positive influence as the coefficient in this case is 0.146 (*P* < 0.001).

**Figure 6:**
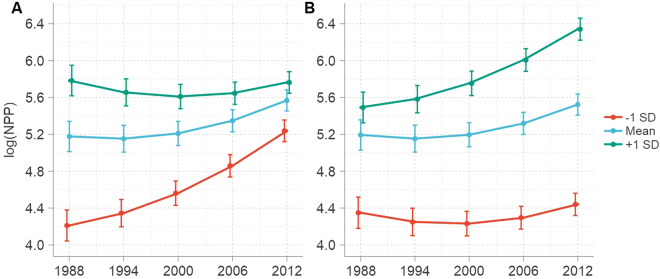
Interactions between time and climatic factors predicting grassland NPP. The curves in this figure were fitted using a conditional three-level growth model (Table S2, Model 6); lines denote log(NPP) trajectories at mean and mean ± 1 SD values of (**A**) precipitation and (**B**) temperature.

We also carried out an additional effect-based analysis of trends in grassland NPP variation, including a series of interaction terms within the three-level nonlinear growth model, time × climate, year × grassland_pct, and socioeconomic development × grassland_pct. These terms were constructed to assess differences in the magnitude of predictors effect as a function of time. Data show that these interactions were significant in terms of climate variables at the watershed scale, with the exception of the relationship between sunlight and time (Table S2). The precipitation-time interaction was also negative for NPP at this scale, indicating that a linear reduction in this latter variable for grasslands was intensified by increased precipitation and that this relationship increases at a faster rate in concert with rainfall levels (Fig. 6A). In contrast, data reveal a positive temperature-time interaction for grassland NPP; this indicates that linear reduction of this latter variable has been weakened by the positive effects of warming and that this relationship changes more rapidly as temperature levels increase (Fig. 6B).

**Figure 7:**
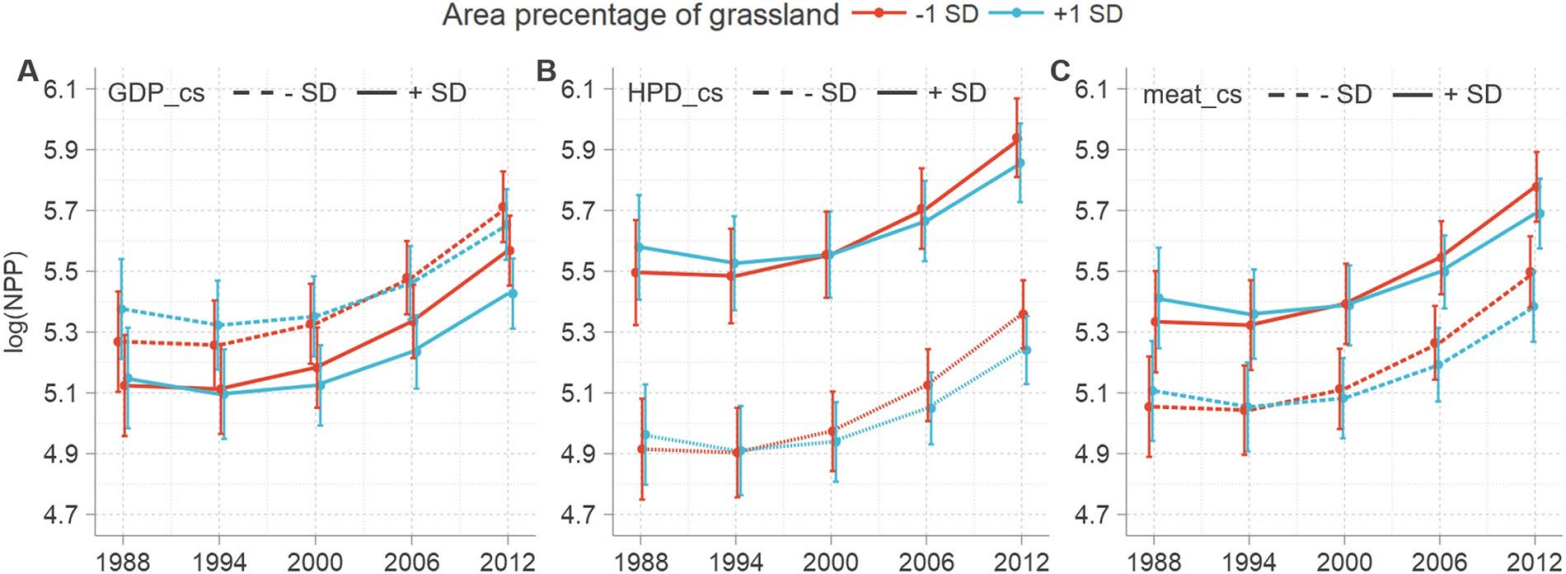
Interactions between grassland area percentage (grassland_pct) and human activity factors predicting NPP. Curves were fitted to a conditional three-level growth model (Table S2, Model 6) and denote the trajectories of log(NPP) at mean ± 1 SD values of (**A**) GDP × grassland_pct, (**B**) HDP × grassland_pct, and **(C)** meat × grassland_pct.

Grasslands are just one important variable that connect socioeconomics to natural ecosystems. Indeed, at the county scale, socioeconomic variables directly impact the area of grassland and thus indirectly influence linear trends in NPP. Data show that GDP-grassland (−0.021, *P* < 0.001) and HPD-grassland (−0.009, *P* < 0.1) interactions both exerted significantly negative effects on the NPP of this ecosystem type, indicating that linear trends in this variable across a watershed characterized by a higher proportion of this land use type tend to be more susceptible to negative economic development and human population growth (Fig. 7). At the same time, the effect of meat production on grassland NPP was positive but non-significant (0.006, *P* = 0.44) over the time period of this study while the area proportion of grassland also directly influences linear trends in productivity. Regression results also show that although grassland area proportion is positively correlated with average NPP, the temporal interaction of the former is negative (−0.003, *P* < 0.001); this means that the rate of increase of grassland NPP will be lower in small watersheds with a high proportion of grassland than in those characterized by smaller areas of this land use type.

## Discussion

### The main sources of grassland NPP variation

The results of this analysis reveal that the most distinct feature of our MLM compared with other longitudinal approaches is the consideration of data in terms of the hierarchical structuring of repeated measures nested within higher level groups^47,48^. This feature means that MLMs can be used to assess variance related to rates of change in individual cases and to test whether, or not, these rates differ depending on the characteristics of higher-level groups^49^. It is clear that grassland ecosystems are multilevel nested structures^50^, and an increasing body of evidence suggests that spatiotemporal variation in NPP within these systems is likely related to direct causes at both meso- and micro-levels (e.g. variability in temperature and precipitation as well as individual vulnerability) as well as indirect effects (e.g. socioeconomic changes at the macro-level)^19,51,52^. The contributions of these factors are likely different, however, at variable scales. The results of this analysis also lend support to this view by combining evidence from both the macro-county level and the small watershed scale (meso-level). Our MLM analysis of grassland NPP changes within the TRHR shows that about 41.1% of variance can be explained at the county level, while the remaining 54.9% occurs at the level of small watersheds. In other words, grassland NPP variation mainly depends on small watershed features; in contrast, the impacts of socioeconomic development and human population growth on grassland NPP variations remain relatively minor.

The impact of climate change and human activities on the grassland NPP in the TRHR. The results of this study demonstrate that precipitation and temperature are the most important climatic factors driving changes in grassland NPP within the TRHR. Previous studies have also recovered similar results and have shown that increased temperature and precipitation are the key factors influencing grassland vegetation growth since the underlying mechanism is significantly increased growing season length^17,53–55^. This analysis also shows that although a ‘warm and moist’ climate trend has been beneficial to increasing grassland NPP, the rate at which this has risen has been faster in relatively drier and warmer regions. This result may be due to the main climatic features of the TRHR; because of the harsh alpine-subalpine environment in this area, total annual precipitation and mean annual temperature remain low during the grassland growth period. This means that although precipitation and temperature are factors that limit plant growth, the spatiotemporal distribution of grassland NPP in the TRHR is nevertheless strongly correlated with both these variables. At the same time, as solar radiation is another key factor determining plant photosynthesis, vegetation growth in small watersheds that are characterized by relatively abundant rainfall and low altitude will also be limited by this variable^56^. A decreased level of solar radiation induced by enhanced precipitation may therefore mitigate the otherwise positive effect of this variable on plant growth; in other words, precipitation and temperature are more beneficial in smaller watershed with lower levels of rainfall and favorable temperatures.

Previous studies have also shown that human activities, including anthropogenic changes to grassland cover, cause major disturbances to alpine grassland ecosystems^43,57–59^. Indeed, since the 1990s, an economic boom in this region has rapidly increased the population level within the TRHR (Fig. S4). Grassland ecosystems have therefore evolved as a consequence of frequent disturbances^60^, especially in middle and eastern areas of the TRHR where intensive human activities have resulted in the over-exploitation of grassland resources^1,61^. The results of this study suggest that economic development and human population growth across the TRHR may have had a negative impact on grassland NPP, while meat production has the opposite effect. These changes might be due to GDP growth (Fig. S4) while increases in population can lead to the conversion of grasslands to cropland and construction land; although these influences will result in degraded grassland^62^, an increasing level of meat production might also encourage herdsmen to pay more attention to grassland management and thus could promote the NPP of these ecosystems^63^. We have also demonstrated that the negative effects caused by human activities are significantly moderated by grassland percentage area; the limitations that result from climatic and topographic conditions mean that human activities within the TRHR are concentrated in central and eastern smaller watersheds which are rich in grassland resources. As these smaller watersheds are therefore subject to more pressure from human activities, the results of this study also support the widespread view that economic development and population levels both harm alpine grassland ecosystems.

### Policy implications for ecological restoration

Grassland ecological restoration is one unique form of human disturbance^64^ that aims to positively mitigate changes due to climate and immigration, maintain ecosystem services, and alleviate poverty^65^. The potential impacts of economic development, increasing population, and livestock production on grassland conversion and growth have provided the focus of international restoration programs^66^. Grassland restoration projects within the TRHR over the last ten to 12 years have been regarded as key responses by the Chinese government, especially since the development of NNRs and the EPRP in 2003 and 2005, respectively. Although the results of this study suggest that the long-term impact of NNRs on grassland NPP has not been significant, the productivity of these ecosystems within the TRHR has nevertheless increased at a faster rate since the initiation of restoration projects and the previous rapid decline has been curbed. This study therefore supports the view that NNRs have had a net positive ecological effect on grasslands.

In contrast to other similar ecosystems, alpine grasslands are mainly distributed within high-altitude zones and cover only about 3% of global land area. These systems are therefore extremely sensitive to human interference^67^; although exploitation and anthropogenic interventions can damage alpine vegetation within just a few seconds, restoration can take several decades^68^ and the available range of approaches that can be applied to such systems remains relatively small^68,69^. At the moment, payments for ecosystem services and governance interventions remain the main ways in which alpine grasslands are restored^65^. Previous research has also shown that both the abiotic and biotic constraints of ecological environments influence plant growth and succession during restoration irrespective of whether short- or long-term tactics are adopted to restore ecosystem resilience and adaptability^70^. Grassland ecosystems within the TRHR provide one representative example of such a system from the high altitude cold environment of the QTP^71^. The results of this study provide clear evidence that vegetation growth and the restoration of alpine ecosystems are severely limited by both abiotic and biotic constraints, including climatic and topographical factors, soil nutrient conditions, and grassland types. The outcomes of this research also imply that the heterogeneity determined by different structures and compositions of abiotic and biotic factors can explain grassland NPP variation. Results imply that large-scale abiotic and biotic changes can substantially influence grassland restoration; measures of heterogeneity should therefore also be incorporated as components of future grassland restoration programs^72^.

## Conclusions

The data presented in this paper show that grassland NPP within the TRHR tended to fluctuate but increase between 1988 and 2012, at an average annual growth rate of up to 1.97%. Changes in grassland NPP reveal that about 41.1% of variance can be explained at the county level, while the remaining 54.9% occurs at the level of small watersheds. Results show that spatiotemporal changes in grassland NPP have mainly been influenced by the distribution of heat and moisture, while precipitation and temperature have been key factors in determining differences in the spatial distribution of grassland quality. Although ‘warm and moist’ trends in climate have been beneficial to increases in grassland NPP, this rate has been faster in relatively drier and warmer regions. In light of these trends, economic development has had a significantly negative effect on grassland NPP growth and this variable has increased at a faster rate in such regions. Economic development and increases in population levels have also had negative impacts on grassland NPP; data suggest that an increase of 149 million CNY and 30.6 thousand people·km^−2^ would likely result in grassland NPP growth decreases of 0.093% and 0.299%, respectively.

## Methods

Spatial variation statistics for vegetation NPP. Spatiotemporal variation in grassland NPP over the study period was analysed using linear regression analyses at the pixel scale. The inter-annual rate of grassland NPP variation can be represented by the slopes of fitted trend lines, expressed as follows:

1 $${\theta }_{slope}=\frac{n\times \sum _{i=1}^{n}i\times NP{P}_{i}-\sum _{i=1}^{n}i\times \sum _{i=1}^{n}NP{P}_{i}}{n\times \sum _{i=1}^{n}{i}^{2}-{(\sum _{i=1}^{n}i)}^{2}}.$$

In this expression, *θ*_*slope*_ denotes the slope of the fitted trend line, n is the research time period, and *NPP*_*i*_ represents vegetation NPP in the *i*-th year. Thus, when *θ*_*slope*_ > 0, the NPP of a pixel shows a decreasing trend over time and otherwise increases. We then tested the degree of confidence in trend variation using the *F* test and graded the slope of trend lines into a series of levels on this basis encompassing an extremely significant decrease (*θ*_*slope*_ < 0 and *P* < 0.01), a significant decrease (*θ*_*slope*_ < 0 and 0.01 ≤ *P* < 0.05), no significant change (*P* ≥ 0.05), significant increase (*θ*_*slope*_ > 0 and 0.01 ≤ *P* < 0.05), and an extremely significant increase (*θ*_*slope*_ > 0 and *P* < 0.01).

### Grassland NPP impact analysis

In order to analyse the impacts of climate change and human activities on grassland NPP, we established a multi-level model encompassing three time levels, small watershed and county scales using classical statistical analysis. Thus, to test the influence of different scales on grassland NPP variation, we initially constructed an empty model that only contained two levels of small watershed and the county scale and which lacked a predictor (Table 2).

**Table 2:** Regression results of the three-level null model.

	Coefficient	Standard error	[95% CI]	
**Fixed parts**				
Intercept	5.361***	0.215	4.939	5.783
**Random parts**				
County level				
τ_00, County_ID_	0.905	0.157	0.644	1.272
Small watershed level				
τ_00, Watershed_ID_	0.977	0.016	0.946	1.009
σ^2^	0.550	0.004	0.542	0.557

Notes: (1) ***, ** and * denote 1%, 5%, and 10% significance levels, respectively; (2) LR test versus MLM: Chi-squared = 19,268.30, *P* > Chi-squared = 0.000.

Resultant Chi-squared test results of the variation components in this empty model show that grassland NPP variation between groups is statistically different to zero; 37.21% of this variation can be decomposed to the county level while 40.17% occurs at the watershed level. Observed grassland NPP therefore encompasses both individual group effects at the level of small watersheds and counties although variation analysis results for grassland NPP based on integrating all data samples may deviate. Thus, to more accurately describe the influence of regional climate change on grassland NPP over time, we introduced a development model encompassing three levels (i.e. time, small watershed, and county) to analyse variation tendencies and the mechanisms that influence grassland NPP. This model was constructed by initially incorporating unconditional three-level growth.

At Level-1, we initially constructed a series of linear and quadratic time terms to examine the temporal relationship of NPP within small watersheds, as follows:

2 $$\mathrm{log}(NP{P}_{tij})={\beta }_{0ij}+{\beta }_{1ij}\times {T}_{tij}+{\beta }_{2jk}\times {T}_{tij}^{2}+{\varepsilon }_{tij}.$$

In this expression, *t*, *i*, and *j* denote time (Level 1), small watershed level (Level 2), and county level (Level 3), respectively. The response log(*NPP*_*tij*_) is therefore log-transformed grassland NPP for a small watershed *i* (belonging to county *j*) at time *t* (*t* = 1, 2, …, *T*), while *β*_0*ij*_ is the intercept item, the first observation of grassland NPP within the small watershed, *j*, when time = 0. Similarly, *β*_1*ij*_ and *β*_2*ij*_ denote the linear and quadratic slopes of grassland NPP within the small watershed, *j*, while the linear slope (*β*_1*ij*_) denotes the instantaneous rate of change at *T*_1_ = 0, and the quadratic slope (*β*_2*ij*_) represents variation in the rate of change. Finally, *ε*_*tjk*_ is residual error, and Var (*ε*_*tij*_) = σ^2^, the variation between the units at the first level.

We then assumed (Level-2) that the intercept and slope of the NPP change trajectory randomly varies amongst small watersheds, and defined Level-2 equations for these terms as follows:

3 $$\begin{array}{rcl}{\beta }_{0ij} & = & {\gamma }_{00j}+{\mu }_{0ij}\\ {\beta }_{1ij} & = & {\gamma }_{10j}+{\mu }_{1ij}\end{array}.$$

In these expressions, *γ*_*00j*_ and *γ*_*10j*_ are the mean intercept and slope pooled over all small watersheds within county *j*, while the residuals *μ*_0*ij*_ and *μ*_1*ij*_ denote the deviation of the intercept and slope around county-specific mean values, respectively. Finally, Var(*μ*_0*ij*_) = *τ*_*β*00_, Var(*μ*_1*ij*_) = *τ*_*β*11_, and *cov* (*μ*_0*ij*_, *μ*_1*ij*_) = *τ*_*β*_; these denote variation between Level 2 units.

The magnitude of specific effects within small watersheds and counties was assumed to vary at Level 3, as follows:

4 $$\begin{array}{rcl}{\gamma }_{00k} & = & {\pi }_{000}+{e}_{00j}\\ {\gamma }_{10k} & = & {\pi }_{100}+{e}_{10j}\end{array},$$

In these expressions, *π*_000_ and *π*_100_ denote the mean intercept and slope which represent average values for grassland NPP pooled over all small watersheds and counties. Thus, *e*_00*j*_ and *e*_10*j*_ denote the deviation of county *j* and the average intercept and slope, while Var(*e*_*00j*_) = *τ*_*π*00_, Var(*e*_10*j*_) = *τ*_*π*10_, and *cov* (*e*_*00j*_, *e*_10*j*_) = *τ*_*π*_ denote variation between Level 3 units.

We then substituted Equation (3) and Equation (4) into Equation (2), as follows:

5 $$\mathrm{log}(NP{P}_{tij})={\pi }_{000}+{\pi }_{100}\times {T}_{tij}+{\beta }_{2jk}\times {T}_{tij}^{2}+{\mu }_{0ij}+{\mu }_{1ij}\times {T}_{tij}+{e}_{00j}+{e}_{10j}\times {T}_{tij}+{\varepsilon }_{tij}.$$

The second stage of model construction involved the incorporation of conditional three-level growth. In this context, vegetation growth is mainly determined by the resultant effects of temperature, solar light, and soil water available to plants^19,38,72–74^. The last of these, soil water availability, is dependent on other environmental factors such as precipitation, topography, and nutrient supply^73,75,76^, while grassland utilization and management as well as indirect socioeconomic activities, such as economic development and population growth, will also all influence vegetation growth^77,78^. A mechanistic model for grassland NPP change should therefore take into account the effects of: (i) Climate factors such as precipitation, temperature, and sunlight; (ii) Soil nutrient supply including the percentage of soil N, P, and K; (iii) Grassland types; (iv) Grassland utilization and management factors including NNRs, the area percentage of grassland, and location, and; (v) Socioeconomic factors such as HPD, GDP, and meat production.

We therefore expanded our unconditional growth model to include a number of predictors. At Level 1, time-varying predictors including climatic (i.e. temperature, precipitation, and sunlight), grassland (i.e. grassland area percentage, types, and NNRs), and socioeconomic factors (i.e. GDP, HPD, and meat production) were entered directly into equations, as follows:

6 $$\mathrm{log}(NP{P}_{tij})={\beta }_{0ij}+{\beta }_{1ij}\times {T}_{tij}+{\beta }_{2jk}\times {T}_{tij}^{2}+{\beta }_{3ij}\times {C}_{tij}+{\beta }_{4ij}\times {T}_{tij}\times {C}_{tij}+{\beta }_{5ij}\times {G}_{tij}+{\beta }_{6ij}\times {T}_{tij}\times {G}_{tij}+{\beta }_{7ij}\times {T}_{tij}\times G{T}_{tij}+{\beta }_{8ij}\times {T}_{tij}\times NN{R}_{tij}.+{\beta }_{9ij}\times {S}_{tij}+{\beta }_{10ij}\times {G}_{tij}\times {S}_{tij}+{\varepsilon }_{tij}$$

In these expressions, *C*_*tij*_ denotes standardized climatic variables, including linear and quadratic functions of temperature, precipitation, and sunlight, while *G*_*tij*_ denotes the standardized grassland area percentage, *S*_*tij*_ denotes standardized socioeconomic factors (i.e. GDP, HPD, and meat production), *GT*_*tij*_ is a categorical variable that denotes the types of grassland within a small watershed (*i*-th), and *NNR*_*tij*_ is a variable factor that notes whether a region is a NNR.

The predictors included at Level 2 are all characteristics of small watersheds, including elevation, slope, and location (i.e. minimum distanced to a water source, highway, and village), as follows:

7 $$\begin{array}{rcl}{\beta }_{0ij} & = & {\gamma }_{00j}+{\gamma }_{0ij}\times {F}_{1ij}+{\mu }_{0ij}\\ {\beta }_{1ij} & = & {\gamma }_{10j}+{\mu }_{1ij}\end{array}.$$

In these expressions, *γ*_01*j*_ denotes the effect of watershed characteristics on the Level 1 intercept, *β*_0*ij*_.

Substituting Equation (7) and Equation (4) into Equation (6) results in:

8 $$\mathrm{log}(NP{P}_{tij})={\pi }_{000}+{\pi }_{100}\times {T}_{tij}+{\beta }_{2jk}\times {T}_{tij}^{2}+{\beta }_{3ij}\times {C}_{tij}+{\beta }_{4ij}\times {T}_{tij}\times {C}_{tij}+{\beta }_{5ij}\times {G}_{tij}+{\beta }_{6ij}\times {T}_{tij}\times {G}_{tij}+{\beta }_{7ij}\times {T}_{tij}\times G{T}_{tij}+{\beta }_{8ij}\times {T}_{tij}\times G{T}_{tij}+{\beta }_{9ij}\times {S}_{tij}+{\beta }_{10ij}\times {G}_{tij}\times {S}_{tij}+{\gamma }_{0ij}\times {F}_{1ij}+{\mu }_{1ij}\times {T}_{tij}+{e}_{10j}\times {T}_{tij}+{\mu }_{0ij}+{e}_{00j}+{\varepsilon }_{tij}$$

We introduce the concept of an ICC within a MLM (Table 3) to explain how much of the proportion of total grassland NPP variation is the result of differences at small watershed and county levels.

**Table 3:** Variance components of the three-level model.

Calculation	Description
$IC{C}_{Watershed\_ID}=\frac{{\tau }_{00,Watershed\_ID}}{({\sigma }^{2}+{\tau }_{00,Watershed\_ID}+{\tau }_{00,County\_ID})}$	Proportion of variance at the small watershed level
$$\begin{array}{l}IC{C}_{County\_ID}=\frac{{\tau }_{00,County\_ID}}{({\sigma }^{2}+{\tau }_{00,Watershed\_ID}+{\tau }_{00,County\_ID})}\end{array}$$	Proportion of variance at county level

### Data sources

Observation points for each variable at the small watershed scale were selected for 1988, 1995, 2000, 2005, 2008, and 2012. Grassland NPP data for each small watershed within the TRHR were obtained from the *Remote Sensing Monitoring Atlas for the Qinghai River Headwaters Ecosystem*^34^, while annual average temperature, precipitation, and hours of sunshine at each time point were extracted from the dataset of *daily climate data from Chinese surface stations for global exchange (V3.0)*^79^. Grassland area proportions at each time point were provided by the Center for Resources and Environmental Sciences, Chinese Academy of Sciences (http://www.resdc.cn)^80^. Our selection of watershed characteristics mainly encompassed the three aspects of topography, soil properties, and location characteristics; thus, elevation and slope were selected as topographic indices, while the percentage contents of N, K, and P were selected as indicative of soil fertility. The soil attribute information used in this study was extracted from the Harmonized World Soil Database V1.2^81^, while average distances between small watersheds and their nearest adjacent highway, water source, and provincial capital were used as location characteristic indices. Categorical variables included grassland types and the presence of NNRs within small watersheds; these data were obtained from the *Remote Sensing Monitoring Atlas for the Qinghai River Headwaters Ecosystem*^34^.

Values for HPD, GDP, and meat production at each time point within counties were extracted from the *Qinghai Statistical Yearbook*^82–87^. Data sources and our pre-processing approach are outlined in Table S3, while the descriptive statistics used for panel data are presented in Table S4.

## Electronic supplementary material

Supplementary material
