# Supplementary material for: Grassland ecosystem responses to climate change and human activities within the Three-River Headwaters region of China
Source: Sci Rep. 2018 Jun 13;8:9079. doi: 10.1038/s41598-018-27150-5 (PMC5998084; doi:10.1038/s41598-018-27150-5)
Supplement: Supplementary file 1 — Supplementary material [file 41598_2018_27150_MOESM1_ESM.docx]

**Grassland ecosystem responses to climate change and human activities within the Three-River Headwaters region of China**

Ze Han^1^, Wei Song^1*^, Xiangzheng Deng^1,2^ & Xinliang Xu^3^

^1^Key Laboratory of Land Surface Pattern and Simulation, Institute of Geographic Sciences and Natural Resources Research, Chinese Academy of Sciences, Beijing 100101, China; hanze1125@163.com (Z.H.); dengxz.ccap@igsnrr.ac.cn (X.D.)

^2^Center for Chinese Agricultural Policy, Chinese Academy of Sciences, Beijing 100101, China

^3^State Key Lab of Resources and Environmental Information System, Institute of Geographical Sciences and Natural Resources Research, Chinese Academy of Sciences, Beijing 100101, China; xuxl@lreis.ac.cn (X.X.)

**Supplementary Materials**

## Changes in land use within the TRHR between 1988 and 2012. Grasslands are the dominant land use type within the TRHR and cover about 67% of total area (Fig. S2). The secondary land use type within this area comprises idle regions that are dominated by gobi and bare rock; these cover more than 23% of the TRHR and are mainly found in high-altitude regions in central and western areas. Forests and waterbodies are relatively uncommon in this part of China, covering less than 5% of total area; the former are mainly distributed within eastern and southern parts of the TRHR, including Nangqian, Xingde, Tongde, Maqin, and Gande counties, while lakes and rivers are scattered throughout high-altitude regions including in Zhiduo and Zaduo counties, in Tunggulashan Town, and in central regions such as in Maduo and Chengduo counties. The accumulated proportions of construction and cultivated land, mainly distributed in eastern counties, both encompass less than 1% of total area.


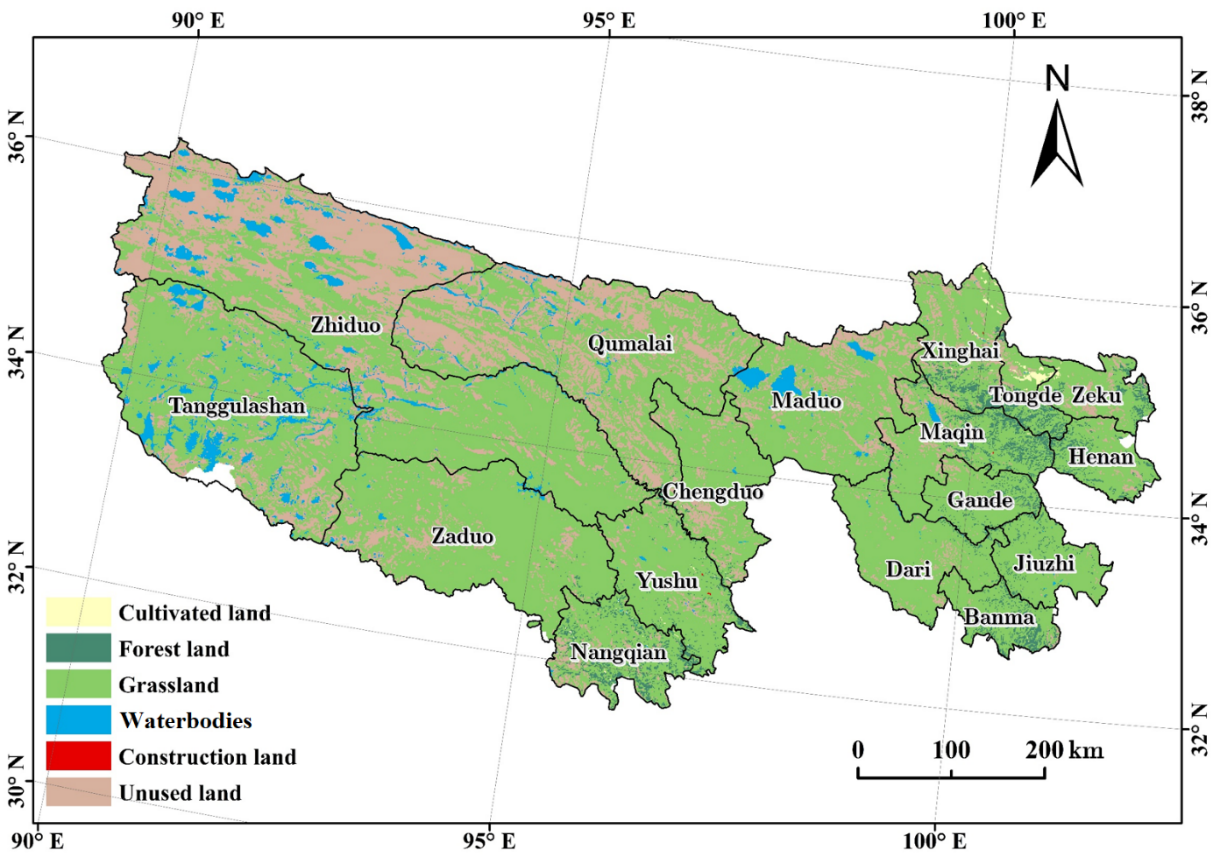


**Figure S1. Map to show land use within the TRHR in 1988.** The land use classification used here is from Liu *et al*.^85^ The map was created using the software ArcMap 10.2.2 (URL: http://www.esri.com/software/arcgis/arcgis-for-desktop)^96^, and the overall dataset was provided by the Data Center for Resources and Environmental Sciences, Chinese Academy of Sciences (http://www.resdc.cn)^95^.

Areas of grassland and unused land within the TRHR were reduced in area by 7,945.2 km^2^ and 246.72 km^2^ between 1988 and 2012, respectively (Table S1). Relative to larger areas, the variation amplitudes of these two land use types are 3.30% and 0.30%, respectively, while waterbodies, cultivated, forested, and construction land areas all increased over this time period. The areas of rivers and lakes had increased by the largest increment by 2012, 5,336.01 km^2^, 31.57% higher than in 1988. Similarly, cultivated land and forested areas were also enlarged by 150.63 km^2^ and 2,704.84 km^2^, respectively, increase amplitudes of about 19.50% in both cases. Changes in construction land area varied slightly at an increase amplitude of just 0.61%.

**Table S1. Changes in land use (hm^2^) within the TRHR between 1988 and 2012.**

|  | | 2012 | | | | | | | | | |
| --- | --- | --- | --- | --- | --- | --- | --- | --- | --- | --- | --- |
|  |  | Cultivated | Forested | Grassland | Water | Construction | | Unused | Transferred-out | | Total |
| 1988 | Cultivated | 33,584 | 5,259 | 33,873 | 2,550 | 646 | 742 | | 43,070 | 76,654 | |
|  | Forested | 5 | 622,338 | 732,396 | 21,896 | 95 | 22,714 | | 777,106 | 1,399,444 | |
|  | Grassland | 55,482 | 978,806 | 17,946,523 | 641,304 | 4,424 | 4,453,040 | | 6,133,056 | 24,079,579 | |
|  | Water | 454 | 5,744 | 380,914 | 992,535 | 203 | 310,188 | | 697,503 | 1,690,038 | |
|  | Construction | 352 | 373 | 4,710 | 290 | 1,194 | 259 | | 5,984 | 7,178 | |
|  | Unused | 1,840 | 57,408 | 4,186,643 | 565,064 | 660 | 3,471,434 | | 4,811,615 | 8,283,049 | |
|  | Transferred-in | 58,133 | 1,047,590 | 5,338,536 | 1,231,104 | 6,028 | 4,786,943 | | 12,468,334 |  | |
|  | Total | 91,717 | 1,669,928 | 23,285,059 | 2,223,639 | 7,222 | 8,258,377 | |  | 35,535,942 | |

Data show that between 1988 and 2012, 35.09% of land area within the TRHR was converted to other uses; the highest transfer rates were seen for grassland, unused land, and areas of water (Fig. S3). Specifically, 6.13 × 10^4^ km^2^ of grassland were transformed over this time period, of which 72.61% was degraded to Unused land mainly in high-altitude southern and western regions, while 10.46% was converted into water notably in northern high-altitude areas such as in Zhiduo County and Tunggulashan Town. Data show that of the 5.34 × 10^4^ km^2^ of grassland that originated from other land use types, 78.42% was originally Unused land located mainly in northern medium- and low-altitude regions such as Zhiduo and Qumarleb counties, while 13.72% was originally forested land in mainly eastern regions such as Maqin, Xinghai, and Gande counties. A total of 7.14% of this transferred land originally comprised areas of water located mainly in the north of Zhidou and Qumarleb counties. Grassland and areas of water encompass 87.01% and 11.74% of transferred-out unused land areas, respectively, and conversion into the latter in this case mainly occurred in the north of Zhidou and Qumarleb counties. At the same time, grasslands and areas of water provided the main transfer-in sources for unused land over this period; degradation of water areas into unused land mainly occurred in western high-altitude zones of the TRHR such as around Tunggulashan Town, while the conversion of cultivated, forested, and construction land only affected relatively small areas in eastern counties. Grassland degradation in these regions was not only the most important direction of transfer-out, but also the main transfer-in land use source, accounting for more than 70% of changes in both directions.


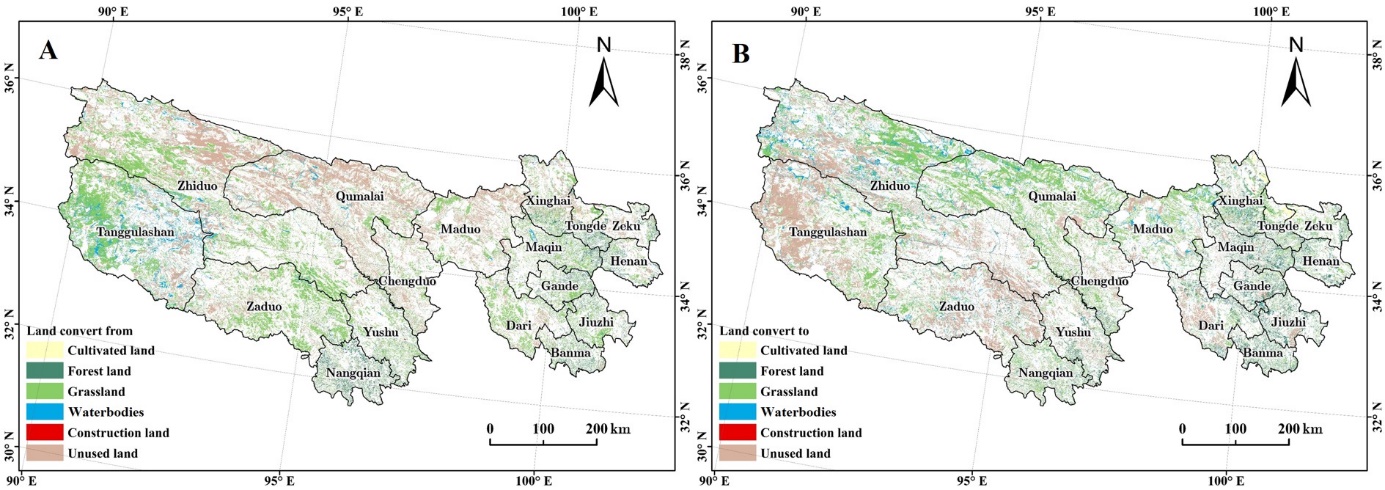


**Figure S2. Maps to show land use type conversions between 1988 and 2012 within the TRHR.** (**A**) 1988 original land use types. (**B**) 2012 final land use types. These maps were created using the software ArcMap 10.2.2 (URL http://www.esri.com/software/arcgis/arcgis-for-desktop)^96^, and the overall dataset was provided by the Data Center for Resources and Environmental Sciences, Chinese Academy of Sciences (http://www.resdc.cn)^95^.


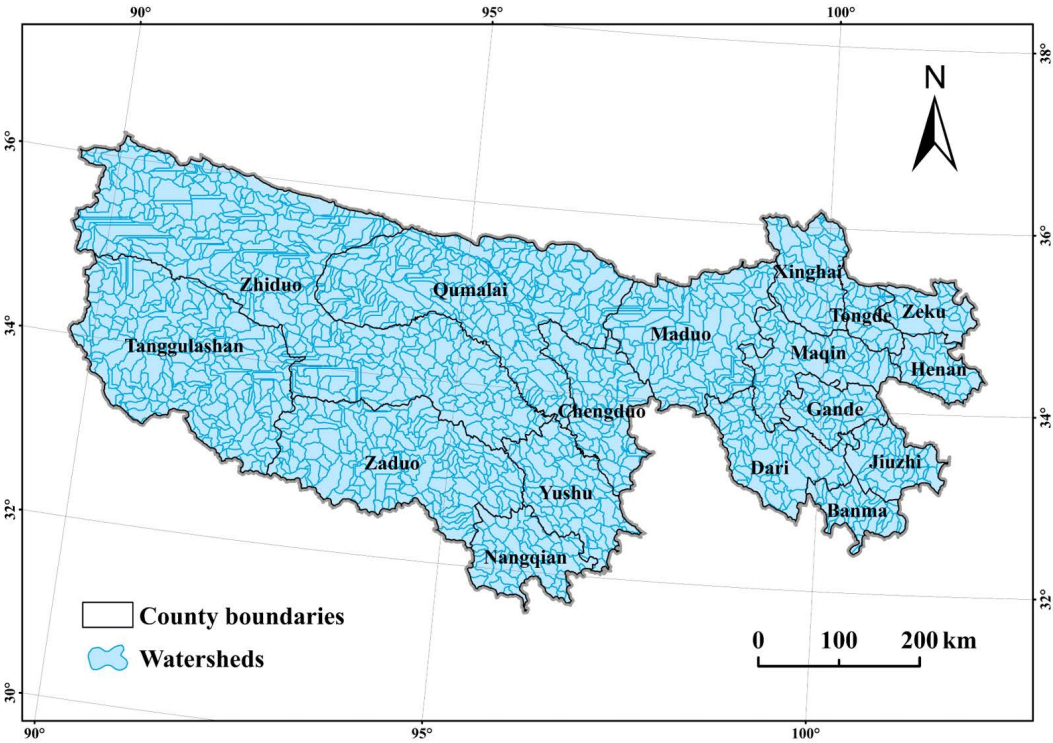


**Figure S3. Map to show the spatial distribution of divided watersheds within the TRHR.** This map was created using the software ArcMap 10.2.2 (URL http://www.esri.com/software/arcgis/arcgis-for-desktop)^96^.

**Table S2. Multilevel model results for grassland NPP changes.**

|  | log(NPP) | | | | | |
| --- | --- | --- | --- | --- | --- | --- |
|  | Model 1 | Model 2 | Model 3 | Model 4 | Model 5 | Model 6 |
|  | *Estimate* (*CI*) | *Estimate* (*CI*) | *Estimate* (*CI*) | *Estimate* (*CI)* | *Estimate* (*CI*) | *Estimate* (*CI*) |
| **Fixed parts** |  |  |  |  |  |  |
| Intercept | 5.135 ***  (4.59 – 5.68) | 5.198 ***  (5.01 – 5.39) | 4.840 ***  (4.18 – 5.50) | 4.610 ***  (3.94 – 5.28) | 4.519 ***  (3.85 – 5.19) | 4.981 ***  (4.25 – 5.71) |
| Year | -0.000  (-0.01 – 0.01) | 0.007  (-0.00 – 0.02) | -0.133 ***  (-0.18 – -0.08) | -0.125 ***  (-0.17 – -0.08) | -0.126 ***  (-0.17 – -0.08) | -0.145 ***  (-0.19 – -0.10) |
| Year^2^ | 0.001 ***  (0.00 – 0.00) | -0.000  (-0.00 – 0.00) | 0.006 ***  (0.00 – 0.01) | 0.006 ***  (0.00 – 0.01) | 0.006 ***  (0.00 – 0.01) | 0.007 ***  (0.01 – 0.01) |
| Rain |  | 0.765 ***  (0.70 – 0.83) | 0.758 ***  (0.70 – 0.82) | 0.841 ***  (0.78 – 0.91) | 0.848 ***  (0.78 – 0.91) | 0.786 ***  (0.73 – 0.85) |
| Rain^2^ |  | -0.293 ***  (-0.33 – -0.26) | -0.269 ***  (-0.30 – -0.23) | -0.256 ***  (-0.29 – -0.22) | -0.253 ***  (-0.29 – -0.22) | -0.180 ***  (-0.21 – -0.15) |
| Temperature |  | 0.783 ***  (0.73 – 0.84) | 0.704 ***  (0.65 – 0.76) | 0.517 ***  (0.44 – 0.60) | 0.525 ***  (0.45 – 0.60) | 0.571 ***  (0.50 – 0.65) |
| Temperature^2^ |  | -0.258 ***  (-0.28 – -0.23) | -0.230 ***  (-0.26 – -0.20) | -0.221 ***  (-0.25 – -0.20) | -0.214 ***  (-0.24 – -0.19) | -0.273 ***  (-0.30 – -0.25) |
| Shour |  | 0.248 ***  (0.18 – 0.32) | 0.254 ***  (0.18 – 0.33) | 0.255 ***  (0.18 – 0.33) | 0.263 ***  (0.19 – 0.34) | 0.162 ***  (0.09 – 0.23) |
| Shour^2^ |  | 0.171 ***  (0.14 – 0.21) | 0.193 ***  (0.16 – 0.23) | 0.189 ***  (0.15 – 0.22) | 0.197 ***  (0.16 – 0.23) | 0.114 ***  (0.08 – 0.15) |
| Year × Rain |  | -0.017 ***  (-0.02 – -0.01) | -0.019 ***  (-0.02 – -0.02) | -0.025 ***  (-0.03 – -0.02) | -0.025 ***  (-0.03 – -0.02) | -0.022 ***  (-0.03 – -0.02) |
| Year × Rain^2^ |  | 0.011 ***  (0.01 – 0.01) | 0.010 ***  (0.01 – 0.01) | 0.009 ***  (0.01 – 0.01) | 0.009 ***  (0.01 – 0.01) | 0.005 ***  (0.00 – 0.01) |
| Year × Temperature |  | -0.003*  (-0.01 – -0.00) | -0.002  (-0.00 – 0.00) | 0.015***  (0.01 – 0.02) | 0.013***  (0.01 – 0.02) | 0.016***  (0.01 – 0.02) |
| Year × Temperature^2^ |  | 0.005 ***  (0.00 – 0.01) | 0.005 ***  (0.00 – 0.01) | 0.005 ***  (0.00 – 0.01) | 0.005 ***  (0.00 – 0.01) | 0.006 ***  (0.00 – 0.01) |
| Year × Sunlight |  | -0.003  (-0.01 – 0.00) | -0.000  (-0.00 – 0.00) | 0.001  (-0.00 – 0.00) | 0.001  (-0.00 – 0.01) | 0.001  (-0.00 – 0.01) |
| Year × Sunlight^2^ |  | -0.009  (-0.01 – 0.01) | -0.009  (-0.01 – 0.01) | -0.009  (-0.01 – 0.01) | -0.009  (-0.01 – 0.01) | -0.007  (-0.01 – 0.00) |
| GT-ASAG |  |  | -0.381  (-1.02 – 0.25) | -0.184  (-0.82 – 0.45) | -0.166  (-0.80 – 0.47) | -0.437  (-1.09 – 0.22) |
| GT-ASAM |  |  | 0.538  (-0.09 – 1.17) | 0.702 *  (0.07 – 1.33) | 0.643 *  (0.01 – 1.27) | 0.398  (-0.26 – 1.05) |
| GT-DG |  |  | -1.486 ***  (-2.14 – -0.83) | -1.288 ***  (-1.95 – -0.63) | -1.141 ***  (-1.80 – -0.48) | -1.521 ***  (-2.20 – -0.84) |
| Year × GT-ASAG |  |  | 0.133 ***  (0.08 – 0.18) | 0.121 ***  (0.07 – 0.17) | 0.119 ***  (0.07 – 0.17) | 0.124 ***  (0.08 – 0.17) |
| Year × GT-ASAM |  |  | 0.140 ***  (0.09 – 0.19) | 0.133 ***  (0.09 – 0.18) | 0.134 ***  (0.09 – 0.18) | 0.136 ***  (0.09 – 0.18) |
| Year × GT-DG |  |  | 0.178 ***  (0.13 – 0.23) | 0.170 ***  (0.12 – 0.22) | 0.168 ***  (0.12 – 0.22) | 0.156 ***  (0.11 – 0.20) |
| Year^2^ × GT-ASAG |  |  | -0.005 ***  (-0.01 – -0.00) | -0.005 ***  (-0.01 – -0.00) | -0.005 ***  (-0.01 – -0.00) | -0.005 ***  (-0.01 – -0.00) |
| Year^2^ × GT-ASAM |  |  | -0.006 ***  (-0.01 – -0.00) | -0.006 ***  (-0.01 – -0.00) | -0.006 ***  (-0.01 – -0.00) | -0.006 ***  (-0.01 – -0.00) |
| Year^2^ × GT-DG |  |  | -0.006 ***  (-0.01 – -0.00) | -0.006 ***  (-0.01 – -0.00) | -0.006 ***  (-0.01 – -0.00) | -0.005 ***  (-0.01 – -0.00) |
| NNRs-Core |  |  | -0.036  (-0.17 – 0.10) | -0.029  (-0.17 – 0.11) | 0.033  (-0.11 – 0.17) | 0.073  (-0.07 – 0.22) |
| NNRs-Buffer |  |  | -0.012  (-0.14 – 0.11) | -0.012  (-0.14 – 0.11) | 0.032  (-0.10 – 0.16) | 0.074  (-0.06 – 0.21) |
| NNRs-Trial |  |  | 0.030  (-0.07 – 0.13) | 0.044  (-0.05 – 0.14) | 0.053  (-0.04 – 0.15) | 0.075  (-0.03 – 0.18) |
| Year × NNRs-Core |  |  | -0.005  (-0.02 – 0.01) | -0.004  (-0.01 – 0.01) | -0.004  (-0.01 – 0.01) | 0.005  (-0.01 – 0.01) |
| Year × NNRs-Buffer |  |  | -0.003  (-0.01 – 0.01) | -0.002  (-0.01 – 0.01) | -0.002  (-0.01 – 0.01) | 0.006  (-0.00 – 0.01) |
| year × NNRs-Trial |  |  | -0.010 **  (-0.02 – -0.00) | -0.011 **  (-0.02 – -0.00) | -0.011 **  (-0.02 – -0.00) | -0.003  (-0.01 – 0.00) |
| Year^2^ × NNRs-Core |  |  | 0.000  (-0.00 – 0.00) | 0.000  (-0.00 – 0.00) | 0.000  (-0.00 – 0.00) | -0.000  (-0.00 – 0.00) |
| Year^2^ × NNRs-Buffer |  |  | 0.000  (-0.00 – 0.00) | 0.000  (-0.00 – 0.00) | 0.000  (-0.00 – 0.00) | -0.000  (-0.00 – 0.00) |
| Year^2^ × NNRs-Trial |  |  | 0.000 *  (0.00 – 0.00) | 0.000 *  (0.00 – 0.00) | 0.000 *  (0.00 – 0.00) | -0.000  (-0.00 – 0.00) |
| Grassland_pct |  |  |  | 0.029  (-0.00 – 0.06) | 0.025  (-0.00 – 0.05) | 0.033 *  (0.00 – 0.06) |
| DEM |  |  |  | -0.234 ***  (-0.33 – -0.14) | -0.101  (-0.20 – 0.00) | -0.108*  (-0.21 – -0.01) |
| Year × Grassland_pct |  |  |  | -0.003 ***  (-0.00 – -0.00) | -0.003 ***  (-0.00 – -0.00) | -0.003 ***  (-0.01 – -0.00) |
| Slope |  |  |  |  | -0.086 ***  (-0.12 – -0.05) | -0.079 ***  (-0.11 – -0.04) |
| Soil_K |  |  |  |  | 0.072 ***  (0.04 – 0.10) | 0.075 ***  (0.04 – 0.11) |
| Soil_N |  |  |  |  | 0.025  (-0.02 – 0.07) | 0.022  (-0.03 – 0.07) |
| Soil_P |  |  |  |  | 0.096 ***  (0.05 – 0.14) | 0.090 ***  (0.04 – 0.14) |
| D2Water |  |  |  |  | 0.032  (-0.00 – 0.07) | 0.031  (-0.00 – 0.07) |
| D2Highway |  |  |  |  | -0.196 ***  (-0.27 – -0.12) | -0.207 ***  (-0.30 – -0.12) |
| D2Village |  |  |  |  | -0.146 ***  (-0.19 – -0.10) | -0.077 ***  (-0.12 – -0.03) |
| GDP |  |  |  |  |  | -0.093 ***  (-0.12 – -0.07) |
| HPD |  |  |  |  |  | -0.299 ***  (-0.37 – -0.23) |
| Meat |  |  |  |  |  | 0.146 ***  (0.12 – 0.17) |
| Grassland_pct × GDP |  |  |  |  |  | -0.021 ***  (-0.03 – -0.01) |
| Grassland_pct × HPD |  |  |  |  |  | -0.009*  (-0.02 – -0.00) |
| Grassland_pct ×Meat |  |  |  |  |  | 0.006  (-0.01 – 0.02) |
| **Random parts** |  |  |  |  |  |  |
| σ^2^ | 0.160 | 0.118 | 0.115 | 0.111 | 0.111 | 0.088 |
| τ_00, Watershed_ID_ | 1.781 | 0.671 | 0.500 | 0.494 | 0.485 | 0.545 |
| τ_00, County_ID_ | 1.354 | 0.149 | 0.154 | 0.143 | 0.178 | 0.445 |
| ρ_01_ | -0.995 | -0.863 | -0.862 | -0.828 | -0.832 | -0.849 |
| N_Watershed_ID_ | 1,817 | 1,817 | 1,817 | 1,817 | 1,817 | 1,817 |
| N_County_ID_ | 18 | 18 | 18 | 18 | 18 | 18 |
| ICC_Watershed_ID_ | 0.541 | 0.715 | 0.651 | 0.661 | 0.627 | 0.505 |
| ICC_County_ID_ | 0.411 | 0.158 | 0.200 | 0.191 | 0.230 | 0.413 |
| Observations | 10,663 | 10,663 | 10,663 | 10,663 | 10,663 | 10,319 |
| R^2^ | 0.932 | 0.953 | 0.954 | 0.956 | 0.956 | 0.965 |
| AIC | 17,554.005 | 13,957.265 | 13,258.664 | 13,216.161 | 13,168.394 | 11,463.034 |

Notes: (1) t statistics in parentheses; (2) ***, **, and * denote 1%, 5%, and 10% significance levels, respectively.

## Study area. Statistical data show that both the economic level and population size within the TRHR has increased annually^87–92^. Records show that both GDP and population increased by 44.36% and 54.88% over this period, respectively; GDP in this calculation was converted into constant prices using 1990 as the base period and the same procedure was followed for other economic indicators. Economic development in this region has been growing rapidly since 2000 (Fig. S4A) even though the overall level of this indicator within the TRHR remains far below the Chinese average and this area remains one of the poorest nationally. The industrial structure of the TRHR region was in a constant state of flux between 1998 and 2012 as primary industry continuously declined and the proportion of secondary industries gradually increased in concert with even slower changes in the tertiary sector. As the TRHR is constrained by unique natural and geographical conditions, this region remains an agricultural zone dominated by animal husbandry with a superimposed primary industrial structure alongside complementary secondary and tertiary industries (Fig. S4B).


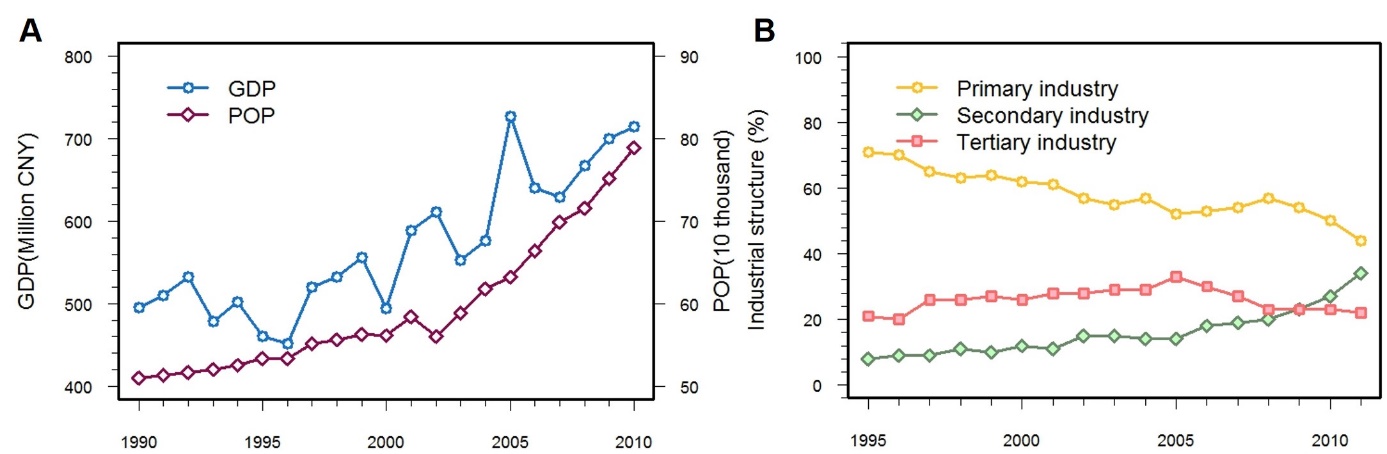


**Figure S4. The socioeconomic situation within the TRHR between the 1990s and 2011.** **(A)** Annual GDP (million CNY) and population (10,000 people) times series across the between 1990 and 2010. **(B)** Time series percentage of industrial sector annual output between 1995 and 2011.

**Table S3. List of** **data sources and preprocessing techniques used in this study.**

| Scale | Data category | Indices | Unit | Data preprocessing technique |
| --- | --- | --- | --- | --- |
| Time scale | Year | 1988, 1995, 2000, 2005, 2008, 2012 | Year |  |
| Watershed scale | Climate | Average annual temperature  Average annual precipitation  Average annual sunshine time | ℃  mm  hour | Monthly average climate data were collected from meteorological observation stations within Qinghai Province and surrounding areas. A thin plate spline approach implemented in the software ANUSPLIN^1^ was then used to perform spatial interpolation to obtain data for each 1,000 m by 1,000 m grid cell |
|  | Vegetation | Average annual NPP | gC·m^-2^·yr^-1^ | Spatial resolution 1,000 m by 1,000 m |
|  | Land cover | Percent of grassland cover | % | Spatial statistical analysis performed using the software ArcGIS 10.2^93^ |
|  | Location | Average distance to the nearest highways  Average distant to the nearest village  Average distant to the nearest water source | km | Spatial statistical analysis performed using the software ArcGIS 10.2^93^ |
|  | Topography | Average elevation of each small watershed  Average slope of each small watershed | M  ° | Slope map generated from a DEM using the software ArcGIS 10.2^93^ |
|  | Soil properties | Average N content in soil  Average P content in soil | % | Statistical analysis performed using the software ArcGIS 10.2^93^ |
| County scale | Social economic | HPD | 10,000 people /km^2^ | Annual GDP was adjusted using the consumer price index |
|  |  | GDP | 10,000 CNY |  |
|  |  | Meat yield | tons |  |

**Table S4.** **Descriptive statistics.**

| *Variable* | *Unit* | *Mean* | *SD* | *Min* | *Max* | *Skewness* |
| --- | --- | --- | --- | --- | --- | --- |
| NPP | gC·m^-2^·yr^-1^ | 228.49 | 179.2 | 0.01 | 909.76 | 0.63 |
| Precipitation | mm | 431.23 | 158.5 | 78.4 | 860.34 | 0.07 |
| Temperature | °C | -3.32 | 2.28 | -10.34 | 4.27 | 0.21 |
| Sunlight | hour | 2,664.42 | 201.05 | 2,151.64 | 3,077.53 | -0.03 |
| Grassland_pct | % | 71.3 | 23.99 | 0.01 | 100 | -0.95 |
| DEM | m | 4,551.83 | 395.18 | 2,965.26 | 5,703.04 | -0.73 |
| slope | ° | 14.07 | 5.8 | 4.67 | 31.08 | 0.74 |
| Soil K | % | 143.51 | 37.17 | 1 | 291.17 | -1 |
| Soil N | % | 0.27 | 0.17 | 0 | 0.81 | 0.61 |
| Soil P | % | 0.06 | 0.01 | 0 | 0.1 | -0.12 |
| D2Water | km | 35.62 | 28.12 | 1.14 | 135.5 | 0.94 |
| D2Highway | km | 802.68 | 196.65 | 348.37 | 1,154.97 | -0.45 |
| D2Village | km | 37.76 | 55.76 | 15.6 | 23.66 | 1.21 |
| GDP | 10,000 | 7,712.82 | 14,864.37 | 1,018.99 | 85,687.65 | 3.91 |
| HPD | 10,000 people·km^-2^ | 2.28 | 3.06 | 0.02 | 16 | 2.22 |
| Meat | ton | 4,360.69 | 2,663.76 | 322 | 18,087 | 1.59 |

# References

1. Hutchinson, M.F. & Xu, T. ANUSPLIN version 4.4 User Guide. Fenner School of Environment and Society, Australian National University, Canberra, http://fennerschool.anu.edu.au/files/anusplin44.pdf (2013).
